# First Record of Isolation and Molecular Characterization of *Aguas Brancas virus*, a New Insect-Specific Virus Found in Brazil

**DOI:** 10.3390/v18020164

**Published:** 2026-01-27

**Authors:** Valéria Cardoso Freitas, Fábio Silva da Silva, Daniel Damous Dias, José Wilson Rosa Junior, Bruna Laís Sena do Nascimento, Maissa Maia Santos, José Leimar Camelo Silva, Ana Raquel Lira Vieira, Ana Cecília Ribeiro Cruz, Sandro Patroca da Silva, Livia Medeiros Neves Casseb, Joaquim Pinto Nunes Neto, Valéria Lima Carvalho

**Affiliations:** 1 Graduate Program in Virology, Evandro Chagas Institute, Health and Environment Surveillance Secretariat, Ministry of Health, Ananindeua 67030-000, PA, Brazil; vallfreittas.vf@gmail.com (V.C.F.); anacecilia@iec.gov.br (A.C.R.C.); liviacasseb@iec.gov.br (L.M.N.C.); valeriacarvalho@iec.gov.br (V.L.C.); 2 Department of Arbovirology and Hemorrhagic Fevers, Evandro Chagas Institute, Health and Environment Surveillance Secretariat, Ministry of Health, Ananindeua 67030-000, PA, Brazil; fabiodasilva@iec.gov.br (F.S.d.S.); damousdias@gmail.com (D.D.D.); josejr@iec.gov.br (J.W.R.J.); brunanascimento@iec.gov.br (B.L.S.d.N.); maissasantos@iec.gov.br (M.M.S.); sandrosilva@iec.gov.br (S.P.d.S.); 3 Directorate of Environmental Health Surveillance, State Secretariat of Health of the Federal District, Brasília 70655-775, DF, Brazil; leimar10silva@gmail.com (J.L.C.S.); ana.raquel@edu.se.df.gov.br (A.R.L.V.)

**Keywords:** *Orthoflavivirus*, Amazon, insect-specific viruses, mosquitoes

## Abstract

Advances in diagnostic techniques, along with environmental changes driven by human activity, have intensified the surveillance and monitoring of virus and arbovirus circulation on the Amazon. These efforts have increased the detection of insect-specific viruses in field-collected hematophagous arthropods. This study reports the first isolation of the *Aguas Brancas virus* from mosquitoes collected in the Brazilian Amazon and in a rural area of Brasília, Federal District, Brazil. Arthropods of the family Culicidae, genus *Limatus durhamii*, were collected at ground level in forest fragments. Sample BEAR812610 originated from Ananindeua, Pará, within the Evandro Chagas Institute’s grounds, and sample BEAR839941 from a forest fragment in Brasília (Ceilândia—Núcleo Rural Boa Esperança, Site B4). Specimens were identified to the species/genus level, macerated, and the supernatant inoculated into C6/36 and Vero cell cultures for viral isolation. The presence of arboviruses was determined by indirect immunofluorescence using antibodies against major arbovirus groups. Positive samples were sequenced for nucleotide and amino acid identification, and phylogenetic analysis confirmed the virus as belonging to the genus *Orthoflavivirus*. This represents the first report of the isolation and characterization of the insect-specific *Aguas Brancas virus*.

## 1. Introduction

Brazil, a country with high levels of biodiversity, has stood out as a focus of study for viral research since the 1950s. Since then, numerous studies have been carried out in which different types of viruses of medical and veterinary importance have been isolated and characterized, as well as some specific insect viruses [1,2,3]. Insect-specific viruses (ISVs) naturally infect mosquitoes and other insects and are able to replicate in insect cells in vitro but not in vertebrate cells. These viruses have been identified in several viral families, including *Flaviviridae*, *Togaviridae*, *Bunyavirales*, *Rhabdoviridae*, *Reoviridae*, and *Mesoniviridae*, reflecting their broad genetic and evolutionary diversity. With advances in molecular biology techniques, particularly high-throughput sequencing, there has been a substantial increase in studies describing ISVs, which have attracted growing interest due to their evolutionary, ecological, and potential biotechnological relevance [4,5,6].

Among ISVs, insect-specific flaviviruses (ISFVs), classified within the genus *Orthoflavivirus* (family *Flaviviridae*), are the most extensively studied group. These viruses possess a single-stranded, positive-sense RNA genome of approximately 11 kb and produce a single polyprotein that is cleaved into three structural proteins (capsid [C], premembrane [prM], and envelope [E]) and seven nonstructural (NS) proteins (NS1, NS2A, NS2B, NS3, NS4A, NS4B, and NS5). Structural proteins are involved in receptor binding, membrane fusion, and virion assembly, whereas nonstructural proteins play key roles in viral RNA replication and modulation of innate immune responses [7,8].

Several studies support the hypothesis that arboviruses may have originated from the evolutionary diversification of ISFVs. Viruses such as *Cell Fusion Agent virus* (CFAV), *Kamiti River virus* (KRV), and *Culex flavivirus* (CxFV), which occupy basal positions in the flavivirus phylogeny, suggest that insect-specific lineages may represent ancestral forms of mosquito-borne flaviviruses [9]. In addition, ISVs have been detected in a wide range of mosquito species, with varying degrees of host adaptation, particularly in *Aedes aegypti*, *Aedes albopictus*, and *Limatus durhamii*. Thus, this study aimed to report the first isolation of *Aguas Brancas virus* in mosquitoes collected in a forest fragment at the Evandro Chagas Institute in Ananindeua, Pará, and Brasília, Federal District.

## 2. Materials and Methods

### 2.1. Mosquito Samples Collection

**Figure 1:**
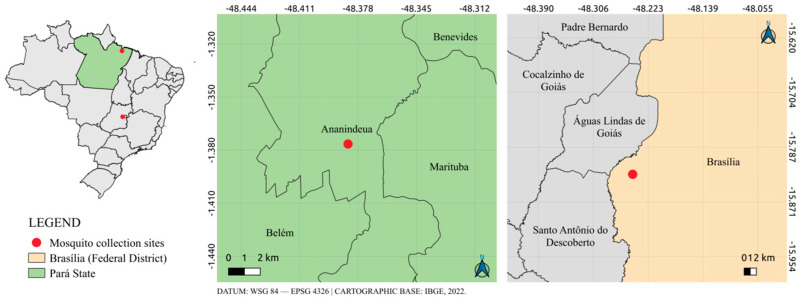
Collection map of mosquito samples used to carry out this study.

The two viral samples described in this study were obtained from mosquitoes of the species *Li. Durhamii*. Each viral sample originated from a distinct pool of *Li. durhamii* mosquitoes and was collected alongside additional mosquito pools comprising other species at two different locations in Brazil. Sample AR812610 was collected in the municipality of Ananindeua, Pará, Brazil, in 2014 from a forest fragment located within the Evandro Chagas Institute’s lands (ground level) as an activity of field research, and sample AR839941 was collected in 2016 from a forest fragment located in Brasília, the capital of Brazil (Boa Esperança Rural Center—Ceilândia) (ground level) from the Epidemiological surveillance staff and subsequently sent to the Evandro Chagas Institute, the Brazilian reference laboratory for arbovirus diagnosis (Figure 1).

The mosquito specimens, collected in a forest fragment located at the Evandro Chagas Institute, were collected using the protected and clarified human attraction technique with the aid of an entomological net and an oral suction trap. The sample collected in Brasília, originated from the investigation of a Yellow Fever outbreak through entomovirological surveillance. After collection, these specimens were transferred to cryovials and transported to the Arbovirology and Hemorrhagic Fevers Section (SEARB/IEC), where they were stored in a freezer at −70 °C until taxonomic identification, which in turn occurred on a refrigerated table at approximately −25 °C. For taxonomic identification, stereomicroscopes (Carl Zeiss, Jena, Germany) were used with the aid of the dichotomous key proposed by Forattini (2002) [10]. Then, the already identified specimens were grouped into batches and labeled with the arthropod registration (BE AR) numbers issued by the SEARB Records. Finally, they were stored at −70 °C for subsequent processing steps.

### 2.2. Routine Laboratory Diagnosis

Viral samples underwent routine diagnostics in the Arbovirology and Hemorrhagic Fevers Section, where they were tested by viral isolation, including specimen maceration, cell inoculation, and indirect immunofluorescence assays. Mosquito pools were macerated in a Tissuelyser equipment (Qiagen, Hilden, Germany) for 60 s at 25 Hz, using 1 mL of 1× D-PBS containing 2% penicillin–streptomycin, 1% Fungizone, and 5% fetal bovine serum (FBS) in 2 mL tubes with a 3 mm tungsten bead. Tubes were frozen at −80 °C overnight, thawed, and centrifuged at 4 °C (10,000 rpm, 10 min) before inoculation [11]. Supernatants (100 µL) were inoculated into C6/36 (*Aedes albopictus*) (ATCC CRL-1660) and Vero (*Chlorocebus sabeus*) (ATCC CCL-81) cell cultures tubes, maintained at 28 °C incubator (L-15 medium with 2% FBS, 1% penicillin/streptomycin, 0,1% fungizone, 1% non-essential amino acids, 2.95% tryptose phosphate) and 37 °C incubator (199 medium with 2% FBS, 1% penicillin/streptomycin, 0.1% fungizone), respectively. Positive and negative controls were included in each test. After one hour of incubation, 1.5 mL of medium was added to each tube with cells, and cultures were observed daily for seven days under an inverted microscope to detect cytopathic effects [11,12]. After viral replication (approximately 7 days), inoculated cells were fixed on slides with analytical-grade acetone (−20 °C) and tested by indirect immunofluorescence assay (IIF). Each slide spot was incubated with 25 µL of in-house–produced polyclonal antibodies diluted 1:20 in phosphate-buffered saline (PBS, pH 7.4), which are routinely used for laboratory diagnosis. Polyclonal antibodies targeting the major arbovirus groups—*Alphavirus*, *Orthoflavivirus*, *Orthobunyavirus*, and *Phlebovirus*—were used for screening. Slides were incubated for 30 min at 37 °C, washed for 10 min in PBS, briefly rinsed in distilled water, and then incubated with 25 µL of fluorescein isothiocyanate (FITC)–conjugated anti-mouse IgG (1:900 dilution; Cappel, Covington, KY, USA) per spot. The same incubation and washing steps were subsequently repeated. Slides were then mounted with buffered glycerol and examined by fluorescence microscopy [13]. Positivity was defined by the presence of specific fluorescence in the cells. Positive and negative controls were included in each assay to validate the results. Samples positive for group B (*Orthoflavivirus*) were further tested using virus-specific antibodies against Yellow fever virus and Dengue virus serotypes 1–4 (Bio-Manguinhos, Fiocruz, Rio de Janeiro, Brazil). Samples that were positive by IIF but could not be identified using virus-specific antibodies were subjected to genome sequencing.

### 2.3. Total RNA Extraction, Sequencing, and Data Processing

A total of 140 µL of cell culture supernatant was used for nucleic acid extraction and purification with the QIAamp Viral RNA Mini Kit (Qiagen). Complementary DNA (cDNA) was synthesized from RNA using the SuperScript™ VILO™ MasterMix kit (Invitrogen, Waltham, MA, USA) for first-strand synthesis and the NEBNext^®^ Second Strand Synthesis Module (New England BioLabs, Ipswich, MA, USA) for second-strand synthesis. The resulting cDNA was purified with the PureLink^®^ PCR Purification Kit (Invitrogen). All procedures followed the manufacturers’ protocols. Purified RNA was used to construct a genomic library with the 454 GL FLX Titanium General Library Kit (Roche, Mannheim, Germany). Fragment quality was verified using a Bioanalyzer, followed by library enrichment via emulsion PCR (emPCR). Sequencing was performed on the GS FLX+ 454 platform (Roche) according to the manufacturer’s instructions.

Raw sequencing reads were processed for quality control using TrimGalore v0.4.5 (available at: https://www.bioinformatics.babraham.ac.uk/projects/trim_galore, accessed on 22 October 2025), removing short reads (<50 nt), adapters, and reads containing >15 undetermined bases (Ns). High-quality reads were assembled de novo with IDBA-UD v1.1.1 [14] and SPAdes v3.12.0 [15]. The resulting contigs were manually inspected in Geneious v9.1.6 [16]. Genomic annotation was performed using DIAMOND [17] against the NCBI non-redundant (NR) protein database, considering e-values < 0.0001 and amino acid identity for taxonomic classification. Predicted coding sequences of the target viral contigs were translated into amino acids and analyzed using InterProScan5 [18] to identify functional protein domains across all available databases.

### 2.4. Phylogenetic Analysis

Phylogenetic inference was performed based on the translation of the complete polyprotein (length with average of 10,177 nt and 3392 aa) (Table S1), as well as the NS3 and NS5 regions of newly obtained sequences and 54 other sequences from different viral species belonging to the genus *Orthoflavivirus* and available in GenBank (NCBI). The nucleotide sequences of the complete polyprotein of the taxa considered in the analysis were initially translated using SeqKit v.2.8.2 [19], then aligned by multiple sequence alignment using the MAFFT algorithm v.7.520 [20], with subsequent manual inspection using Aliview v.1.28 [21]. Then, using IQ-TREE v.1.6.12 [22], the best amino acid substitution models (LG+F+R8 for complete polyprotein, LG+F+R6 for NS3, and LG+R7 for NS5) was determined according to the Akaike information criterion (AIC). Subsequently, the phylogenetic signal was evaluated [23], and the phylogeny was reconstructed using the Maximum Likelihood method, with support values (bootstrapping) set to 1.000 pseudoreplicates. Finally, the obtained topology was visualized using FigTree v.1.4.4 (available at: https://tree.bio.ed.ac.uk/software/figtree, accessed on 22 October 2025), with rooting from the midpoint (midpoint rooting) of distances statistically calculated by the software.

## 3. Results

### 3.1. Initial Diagnosis by Routine Laboratory Tests

The samples analyzed in this study originated from epidemiological surveillance (Brasília) and field research (Ananindeua). They were processed together with other samples from the same locations and inoculated into C6/36 and Vero cell lines. No cytopathic effect (CPE) was observed in either culture. Despite the absence of CPE, the samples were tested by indirect immunofluorescence (IIF) for several viral groups, including arboviruses. Samples AR812610 and AR839941, obtained from *Limatus* mosquitoes, tested positive for group B arboviruses (*Orthoflavivirus*) by IIF. Subsequent testing for the four *Dengue virus* serotypes (DENV-1, DENV-2, DENV-3, and DENV-4) and for the *Yellow fever virus* (YFV) yielded negative results. As viral typing was not possible by IIF, these isolates were submitted for genomic sequencing to identify the viral agent.

### 3.2. Viral Genomes Obtained

**Figure 2:**
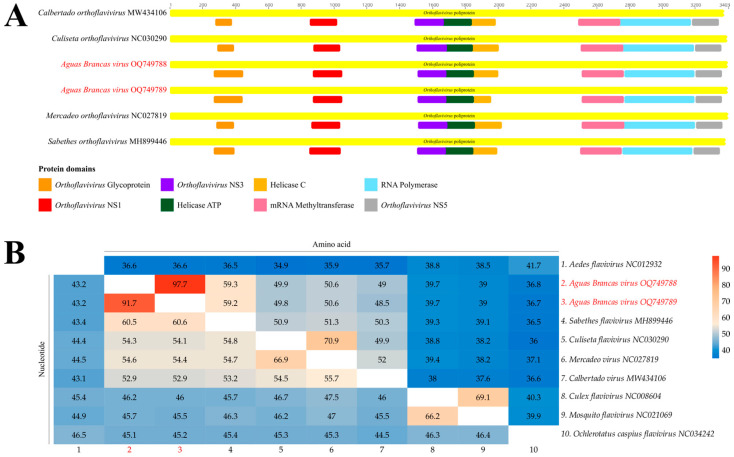
(**A**) Representation of the *Aguas Brancas virus* polyprotein functional domains, comparing the identified contigs with close sequences. (**B**) Heatmap of the polyprotein’s nucleotide (lower triangle) and amino acid (upper triangle) identities of the three study sequences and close sequences.

Based on the data generated by sequencing of the investigated samples, it was possible to completely recover the coding region of two strains related to a single, potentially new viral species of the *Orthoflavivirus* genus, presenting conserved domains similar to other species in the group (Figure 2A) and average nucleotide and amino acid identities of 60.5% and 59.3%, respectively (Figure 2B). The nucleotide sequences were deposited in GenBank (NCBI) under accession codes OQ749788 (AR812610, collected in Ananindeua, Pará State) and OQ749789 (AR839941, collected in Brasília), presenting average coverages of 27.6x and 33.9x, respectively.

**Figure 3:**
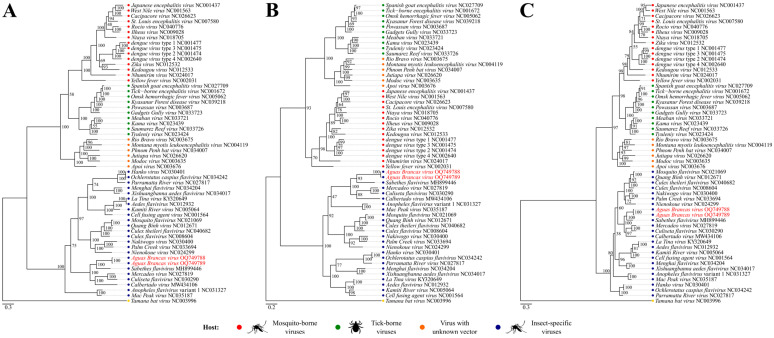
Phylogenetic reconstruction based on the amino acid sequences of the *Aguas Brancas virus* and other 54 taxa available on GenBank. (**A**) Complete polyprotein region. (**B**) NS3 region. (**C**) NS5 region. Bootstrapping support values (BS) are shown on each node. The colored dots indicate the main reconstructed taxonomic groupings. The obtained sequences are highlighted in red.

Phylogeny reconstructions resulted in topologies with high internal anchoring values and composed equally of four well-established monophyletic clusters (Figure 3), with an average support of 98.2% of resolved quartets after phylogenetic signal analysis of the evaluated marker sets. The topologies clearly demonstrated the division of clades according to known hosts, being labeled and identified as: Mosquito-borne viruses (BS = 70 ± 100%), Tick-borne viruses (BS = 100%), Viruses without known vector (BS = 100%), and Insect-specific viruses (BS = 100%). The investigated taxon, in all analyses, showed greater proximity to the taxon *Sabethes flavivirus* (GenBank ID: MH899446) (BS = 93 ± 100%), and is included in the latter clade.

## 4. Discussion

This study described and characterized two potential strains for insect-specific orthoflavivirus genomes belonging to the genus *Orthoflavivirus*, with tentatively named *Aguas Brancas virus*. The viruses were obtained from *Li. durhamii* mosquitoes in two geographically distinct regions of Brazil: Ananindeua, Pará state (northern region), and Brasília, Federal District (central-western region). This identification contributes to the growing number of known insect-specific viruses (ISVs), a group recognized for its wide diversity, cosmopolitan distribution, and potential to infect various mosquito and sandfly species. The evolutionary relationship between ISVs and arboviruses remains an important area of investigation, as ISVs may represent ancestral lineages from which some arboviruses evolved [4].

Analysis of conserved functional domains demonstrated that the potential novel flavivirus maintains a typical polyprotein architecture observed in members of the *Orthoflavivirus* genus [24,25], particularly based on the main domains associated with non-structural proteins, including the helicase in NS3 and the RNA-dependent RNA polymerase in NS5. The conservation of these domains suggests that such regions are under strong purifying selective pressure, since they play essential roles in polyprotein processing and viral genome replication [26,27]. On the other hand, variations observed outside these core regions may reflect adaptive processes associated with differences in host, vector, or viral ecology, as previously described for emerging and divergent flaviviruses [28].

Additionally, the reconstructed phylogenies, inferred from the complete polyprotein and the NS3 and NS5 regions, reveal congruent topologies, with the new flavivirus occupying a consistent position and closely related to other ISVs across the different analyses. The use of these regions as robust phylogenetic markers is widely established for flaviviruses, particularly NS5, one of the most conserved regions of the genome [27]. The concordance between the obtained topologies further suggests the existence of a stable evolutionary signal throughout the viral genome, supporting the phylogenetic classification proposed for the virus investigated in this study.

The *Li. durhamii* samples from Pará were collected in a forest fragment surrounded by urban areas with intense human activity, while those from Brasília came from a more rural environment. The occurrence of these viruses in anthropogenic areas raises the hypothesis that *Li. durhamii* may adapt to human-influenced habitats. This finding highlights the potential for the emergence of novel pathogens and underscores the influence of human activity on viral ecology. Additionally, little is known about the pathogenicity or ecological behavior of *Aguas Brancas virus*. Continuous surveillance and vector monitoring remain essential for arbovirus prevention and control. Previous studies have identified *Limatus* spp. and *Wyeomyia* spp. as potential vectors of Amazonian pathogens [29,30]. Barrio-Nuevo et al. (2020) [31] also detected Zika virus (ZIKV) in *Li. durhamii* mosquitoes in São Paulo, suggesting their possible involvement in arbovirus transmission. However, further studies are needed to confirm the vector competence of this species for ZIKV and other pathogens. In this context, future investigations should focus on the interaction between *Li. durhamii* mosquitoes and insect-specific viruses, aiming to elucidate their ecological relationships, vector competence, and potential role in arbovirus evolution and maintenance in nature [31,32,33,34].

## Data Availability

All the data obtained during this study are available on the tables and figures included in the text. The sequences of *Aguas Brancas virus* obtained here were deposited in the GenBank database under accession codes OQ749788 and OQ749789.

## Supplementary Materials

The following supporting information can be downloaded at: https://www.mdpi.com/article/10.3390/v18020164/s1.
